# Health effects of intermittent fasting and the role of artificial intelligence technologies in optimizing its clinical translation

**DOI:** 10.3934/publichealth.2025061

**Published:** 2025-12-18

**Authors:** Chenghao Zhang, Lijun Chang, Yanqiu Huang, Yadan Xu, Wen Gu, Yang Yang, Hui Wang

**Affiliations:** School of Public Health, Shanghai Jiao Tong University School of Medicine, Shanghai, 200025, China

**Keywords:** intermittent fasting, artificial intelligence, chronic disease prevention, nutrition, multi-omics

## Abstract

As a non-pharmacological approach, Intermittent Fasting (IF) exhibits the capacity to boost health and counteract chronic diseases by regulating the metabolism, strengthening the cellular resistance to stress, and reshaping the immune microenvironment. The rapid progress of Artificial Intelligence (AI) technologies has greatly advanced our comprehension of IF's diverse health benefits. This review outlines AI's role in enhancing the exploration of IF's function in governing systemic health, clarifies the association between IF and health outcomes, and specifies AI's function in analyzing IF's impacts, which cover metabolic processes, cellular stress responses, disease prevention, and the development of personalized dietary strategies. By leveraging AI to integrate various omics datasets, the mechanisms through which IF prevents chronic diseases can be uncovered. This review discusses the challenges that AI faces in researching diet-related health mechanisms and presents an outlook on future developments. AI offers innovative methods to investigate IF's effects on chronic disease prevention, which could lay the foundation for more efficient strategies to support healthier and longer lifespans.

## 1. Introduction

Chronic non-communicable diseases (NCDs) represent one of today's most pressing global health challenges. These conditions not only diminish an individual's quality of life but create substantial economic burdens for individuals and nations alike in prevention and treatment costs. The global impact of NCDs has dramatically grown in recent years. Between 2010 and 2023, the total burden measured in disability-adjusted life years (DALYs) surged from 1.45 billion to 1.8 billion [1]. The human toll is equally staggering. In 2021 alone, NCDs accounted for approximately 43 million deaths, representing 75% of all non-epidemic-related fatalities. Of particular concern are the roughly 18 million premature deaths (in individuals under 70 years of age), with 82% occurring in low- and middle-income countries [2]. Among the world's one billion people living in extreme poverty, NCDs impose a substantial yet frequently overlooked burden [3]. China's situation highlights these global disparities: while home to 17.9% of the world's population in 2021, the country accounted for 25.9% of global NCD-related deaths [1]. In response to this complex landscape, the Global Alliance for Chronic Diseases (GACD) continues to coordinate tailored prevention efforts across different regions [4].

Robust scientific evidence consistently demonstrates that healthy eating patterns play a crucial role in preventing chronic diseases [5]–[7]. This understanding has shifted the focus of chronic disease prevention toward innovative dietary approaches. Several evidence-backed dietary regimens, including the Dietary Approaches to Stop Hypertension (DASH) diet, the Alternative Healthy Eating Index 2010 (AHEI–2010), the Healthful Plant-based Diet Index (hPDI), and the Planetary Health Diet Index (PHDI), have shown distinct health benefits [8]–[16]. Within this context, caloric restriction (CR) has emerged as a fundamental intervention strategy. By reducing the daily energy intake by 20%–40% while maintaining proper nutrition, CR activates key pathways such as AMPK/SIRT1, enhances mitochondrial function, and reduces oxidative stress. A review of CR suggests that these biological responses collectively contribute to the prolongation of healthy lifespans in multiple model organisms [17].

Building on CR principles, Intermittent Fasting (IF) has gained attention for its approach to metabolic regulation. Rather than continuous CR, IF restructures eating patterns to create fasting cycles that induce mild metabolic stress. This triggers adaptive responses including autophagy activation and stem cell regeneration [18],[19]. Current IF approaches primarily include Time-Restricted Eating (TRE), Fasting-Mimicking Diets (FMD), Alternate-Day Fasting (ADF), and the 5:2 Diet. While the implementation protocols vary across different IF regimens, all have demonstrated efficacy in weight management and physiological homeostasis [18],[20], a finding corroborated by evidence that diverse execution strategies yield comparable benefits for weight reduction and physiological regulation [17]. Although IF yields short-term fat loss and weight reduction on par with conventional dietary interventions, its long-term health sequelae remain incompletely characterized, including the risk of lean mass loss linked to unsupervised IF implementation, a concern that is particularly pronounced in elderly populations. In this demographic, IF-associated lean mass loss may accelerate sarcopenia, thereby exacerbating functional decline and elevating comorbidity burden. Thus, the scientific community continues to debate these aspects, thus underscoring the need for large-scale, multi-center, long-term studies that can fully elucidate IF's sustained health effects and underlying mechanisms.

Recent technological advances are helping to address these questions. Multi-omics technologies provide powerful methodologies to decipher the molecular basis of IF-related health benefits. Meanwhile, Artificial Intelligence (AI), which has demonstrated remarkable data analysis capabilities across multiple medical fields, offers promising new directions for developing personalized IF programs [21]. AI integration in digital pathology has significantly improved the image analysis accuracy and efficiency, thus enabling automatic tumor detection, biomarker identification, and treatment response prediction [22]. Driven by demands for high-throughput analyses, automated deep learning (DL) models, particularly convolutional neural networks (CNNs), can accurately diagnose cancer, classify subtypes, and determine tumor grade from pathological specimens [23]. AI's potential has already been validated in personalized nutrition [24], with a clinical study comparing the efficacy between AI-driven diets and traditional diets, where AI-based personalized microbiome modulation through diet significantly improved irritable bowel syndrome-related symptoms [25].

This review aims to synthesize evidence from multi-omics and multimodal data sources to explore IF's regulatory effects and mechanisms. It highlights AI's unique utility in elucidating how IF may prevent chronic diseases, ultimately bridging methodological innovation with biological insight to advance this evolving field.

## 2. Health effects of intermittent fasting

### 2.1. Overview of research on the health effects of intermittent fasting

As a non-pharmacological intervention, IF is perhaps best known for its ability to reduce body weight and body fat. Substantial research demonstrates that IF can positively regulate numerous obesity-related metabolic parameters. An Umbrella review of meta-analyses of randomized clinical trials demonstrated that IF consistently delivers notable benefits in individuals with obesity-related metabolic disorders, thereby reducing body mass index (BMI), body fat percentage, and visceral adipose tissue accumulation while simultaneously improving glucose homeostasis and insulin sensitivity [26].

**Figure 1.**
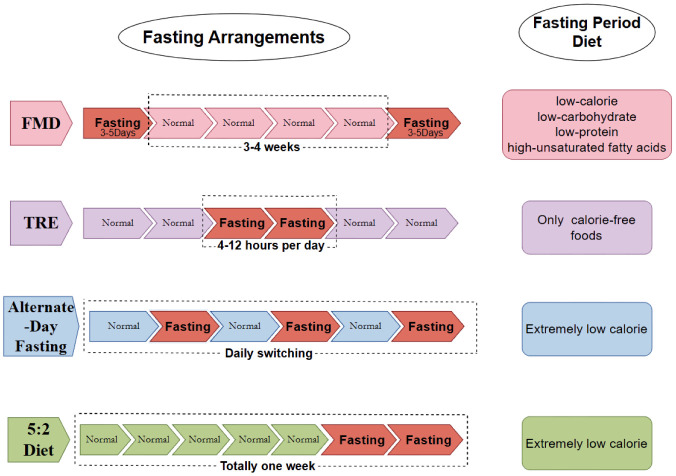
Overview of the classification and operation methods of Intermittent Fasting. FMD aims to “induce” the body into a fasting state based on meeting the body's most basic needs through low-calorie, low-protein, low-carbon, high-unsaturated fat diets. TRE involves fasting by limiting the daily eating window to 4–12 h and consuming almost no calorie-containing foods during the rest of the day. ADF involves fasting or inducing a very low-calorie intake every other day, normal eating on non-fasting days, or a daily cycle of alternating practices. For the 5:2 diet, on a weekly basis, the individual has a normal diet for 5 days and an extremely low-calorie intake for 2 days.

Current approaches primarily include TRE, ADF, and the 5:2 Diet, each with unique characteristics [27], as illustrated in Figure 1. TRE typically involves confining the eating window to 4–10 h and fasting for the remaining hours of the day (14–20 h fast). During the eating window, individuals are not required to count calories or monitor food intake in any way. During the fasting window, individuals are encouraged to drink plenty of water or may also consume energy-free beverages [28]. FMD achieves the body's most fundamental requirements through a diet low in calories, protein, carbohydrates, and unsaturated fats. This approach ‘induces’ the body into a fasting state [29]. As nutritional needs are met, this form of fasting can be sustained for longer periods compared to other fasting regimens, thus allowing for complete fasts of 3–5 days each month. ADF, as its name implies, consists of one day of normal eating followed by a fasting schedule with very low or no caloric intake [30]. The 5:2 diet is a periodic fasting regimen that involves a very-low-calorie diet for 2 days per week and a regular diet for the remaining 5 days [20]; based on the 5:2 diet, some teams are also attempting to alter fasting rhythms on a weekly cycle, such as 1:6, 4:3, or other ratios that allocate periods of fasting and unrestricted eating. Compared to the ADF, the 5:2 Diet better aligns with the lifestyle of the typical working staff, thereby allowing fasting to be undertaken during the more relaxed weekend period.

Despite some similar effects of IF, different models have different physiological effects. We compiled a tabular summary detailing the health effects of IF in animal studies and human trials, which facilitates researchers in comparing the similarities and differences of IF's health effects between different organisms (Tables 1 and 2). For instance, a review showed that short-term (4–12 h per day for 4–12 weeks) TRE enhanced metabolic function partly through circadian rhythm modulation in both human and animals, though long-term (half and even one year) adherence remains challenging [31]. In patients with non-alcoholic fatty liver disease (NAFLD), combining ADF with exercise for 3 months significantly reduced the intrahepatic triglyceride content and body weight, and improved the insulin sensitivity and glucose metabolism [32]. Meanwhile, 8-week human trials indicated that the 5:2 Diet promotes greater weight loss than conventional healthy diets, an effect potentially influenced by apolipoprotein E and SLC16A7 genotypes. These findings also suggest possible cognitive benefits, thus pointing to a beneficial role for the 5:2 Diet in brain health [33]. Researchers continue to refine IF protocols through innovative models. One 3-month clinical trial randomized 108 overweight individuals into early or late TRE combined with energy restriction. The early TRE group demonstrated superior improvements in body fat percentage, diastolic blood pressure, metabolic age, and fasting glucose compared to both late TRE and CR-only groups. This underscores the importance of “temporal specificity” in personalizing metabolic interventions [34].

**Figure 2.**
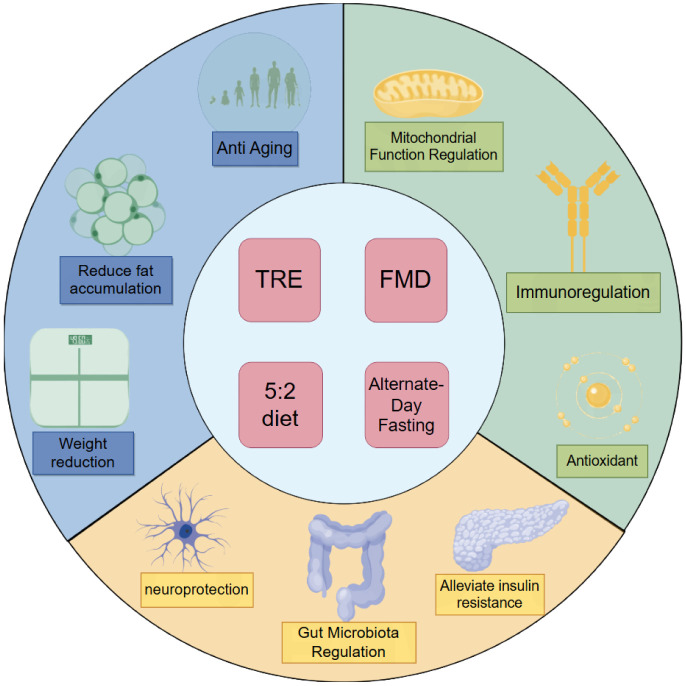
Brief review of the molecular mechanisms underlying the health effects of Intermittent Fasting. The sources of health effects of IF are rich. At the macro level, IF can control the BMI and reduce fat accumulation and anti-aging. At the tissue level, IF can reduce insulin resistance by reducing neuroinflammation, thus remodeling gut microbiota. In terms of molecular mechanisms, IF can also improve the mitochondrial function and regulate the immune ability, anti-excessive oxidation, and anti-free radicals.

Collectively, these studies establish that IF exerts multifaceted health benefits (Figure 2), particularly in alleviating the burden of metabolism-related diseases. The accumulating evidence strongly supports IF as a valuable therapeutic strategy worthy of continued investigation.

**Table 1.** Health effects of different IF protocols in human trials.

Diet type	Fasting period	Protocols	Object	Health effects
FMD	7–10 days	medical short-term fasting	Healthy person	Reduces disease activity (measured by DAS28 scores) and inflammatory markers including CRP while alleviating joint symptoms
	3–9 months	low in calories, sugars, and protein but high in unsaturated fats	Healthy person	Reduced body weight, trunk, and total body fat; lowered blood pressure; and decreased insulin-like growth factor 1 (IGF-1) after 3 cycles, the post hoc analysis of FMD showed that body mass index, blood pressure, fasting glucose, IGF-1, triglycerides, total and low-density lipoprotein cholesterol, and C-reactive protein were more beneficially affected in participants at risk for disease than in subjects who were not at risk
	8 consecutive cycles (average complete 5.5 to 6.8 cycles)	5-day plant-based, low-calorie (600 kcal on day 1, followed by 300 kcal/d on days 2–5), low-protein, low-carbohydrate diet (patent pending) and repeated every 3 or 4 weeks	patients with hormone-receptor-positive breast cancer	Cause metabolic changes analogous to those observed in mice, including reduced levels of insulin, leptin and IGF1, with the last two remaining low for extended periods
TRE	3-month	16 hours fasting per day	Healthy person	Improve body fat percentage, diastolic blood pressure, metabolic age, and fasting glucose
	6-week	16 hours fasting per day	healthy young men	Reductions in body weight and fat mass, along with increased basal fat oxidation
	6-month	20 hours fasting on three nonconsecutive days per week	type 2 diabetes patients	Improve glycated hemoglobin and insulin sensitivity
	2-week	16 hours fasting per day	Resistant Hypertension (RH) patients	Reduced blood pressure in RH patients, Accompanied by a shift in the gut microbiota, including increased abundance of *Akkermansia muciniphila* and *Adlercreutzia equolifaciens*
	4-week	dawn-to-dusk (15 hours fasting per day)	overweight works individuals	Upregulated key autophagy genes (LAMP2, LC3B, and ATG5) and improved metabolic and inflammatory markers in overweight individuals
	3-week	16 hours fasting per day	obese type 2 diabetes patients	Reduced LDL cholesterol, total cholesterol, and leptin levels while elevating β-hydroxybutyrate
	72-hour fasting		Healthy person	Triggered extensive transcriptomic and proteomic changes in white blood cells, upregulated autophagy pathways while downregulating apoptosis and enhanced leukocyte viability and stimulates neutrophil degranulation and cytokine secretion
	30-day	16 hours fasting per day	Healthy person	Ameliorated immunosenescence and promoted gut microbiota health by modulating CD4⁺ Treg cells and increased *Akkermansia muciniphila* abundance
ADF	3-month		non-alcoholic fatty liver disease	Reduced intrahepatic triglyceride content, body weight, and improved insulin sensitivity and glucose metabolism
	4-week		individuals with obesity	Improved the glucose tolerance, effects linked to gut microbiota changes (including *Alistipes finegoldii*) and short-chain fatty acid production
5:2 Diet	8-week		older adults aged 55-70 years	Influenced by apolipoprotein E and SLC16A7 genotypes, greater weight loss than conventional healthy diets
	24-week		overweight or obese shift workers	Led to significant weight loss and improved insulin resistance (measured by HOMA-IR) and achieved greater reductions in total cholesterol and LDL cholesterol
	12-week	3-day per week	among insulin-treated type 2 diabetes patients	Achieved sustained weight reduction over a two-year follow-up and demonstrated excellent safety

**Table 2.** Health effects of different IF protocols in animal experiments.

Diet type	Fasting period	Protocols	Object animal	Health effects
FMD	42-day	On day 1 of the 50% calorie restriction. On days 2–3 of the 10% CR diet. On days 4–7 provided ad libitum.	AOM+DSS mouse CRC models	Enriched with *Bifidobacterium pseudolongum*, which promoted the differentiation of CD8+ T cells into memory cells by generating L-arginine, thereby synergistically demonstrating the anti-tumor effect of oral decisive therapy on colorectal cancer
	42-day	The day 1 low caloric intake. The day 2–4 extremely low-calorie intake. Then 10 days fed ad libitum	mouse breast cancer model	Delayed the progression of breast cancer and melanoma by increasing the number of bone marrow common lymphoid progenitor cells and cytotoxic CD8+ tumor-infiltrating lymphocytes and, at least in a mouse breast cancer model, the downregulation of the stress-responsive enzyme heme oxygenase-1
	35-60 days	8–96 hours every week	mouse models of hormone-receptor-positive breast cancer	Enhanced the activity of the endocrine therapeutics tamoxifen and fulvestrant by lowering circulating IGF1, insulin and leptin and by inhibiting AKT–mTOR signaling via upregulation of EGR1 and PTEN
TRE	8-week	16 hours fasting per day combined with physical exercise	rats	Produced optimal outcomes in androgen regulation and muscle development
	10-week	18 hours feeding per day	aged mice	Enhanced motor coordination and muscle strength while increasing myelin-associated glycoprotein expression
	3-month	18 hours fasting per day	high-risk AD mice model	Enhanced brain-derived neurotrophic factor signaling constitutes one mechanism through which fasting interventions ameliorate cognitive decline
	5-week	16 hours fasting per day	diabetes-prone mice model	Substantial alternative splicing events in skeletal muscle, differentially regulating metabolic pathways through altered enzyme activities and intracellular signaling, collectively optimizing energy utilization
	2-week	16 hours fasting per day	drug-resistant spontaneously hypertensive rats (SHRs)	Fecal microbiota transplantation (FMT) from drug-resistant SHRs successfully transferred both hypertension and impaired drug efficacy to recipient rats. Supplementation with *Akkermansia muciniphila* and *Adlercreutzia equolifaciens* significantly lowered blood pressure in SHR rats resistant to antihypertensive drugs
	36-day	16 hours fasting per day	dextran sodium sulfate (DSS)-induced colitis mice model,	Reduced intestinal oxidative stress by decreasing intracellular reactive oxygen species while enhancing antioxidant enzymes including superoxide dismutase (SOD) and glutathione peroxidase (GSH-Px)
	4-week	12 hours fasting per day	males and females mice	Reversed high-fat diet-induced memory impairment by restoring normal cortex-hippocampus neural coupling, linked to rebalanced glucocorticoid receptor Ser134/Ser226 phosphorylation
	6-month	12-16 hours fasting per day	mice	Enhanced cardiac energy metabolism efficiency via AMPK activation and remodeled intracellular mitochondrial networks to support heart function
	3-week	12 hours fasting per day	Per1^−/−^ Per2^−/−^ mice	Synchronized mitochondrial metabolic rhythms through clock genes such as CLOCK/BMAL1, resulting in remarkable 100% improvements in exercise endurance
	22-mouth	16 hours fasting per day	mice	Improved metabolic homeostasis by enriching SCFA-producing bacteria (including *Akkermansia muciniphila*) and regulated key polyunsaturated fatty acid metabolic pathways
	7-week	15 hours fasting per day	mice	Enhanced the rhythm of gene expression in most tissues, integrated the expression of anabolic and catabolic genes, down-regulated the inflammatory signaling and triglyceride metabolism related genes, and up-regulated the RNA processing, protein folding and autophagy related genes.
	45-week	caloric restriction combined with 20 or 16 hours fasting per day	adult female mice	Altered serum metabolic profiles in aged female mice, reduced oxidative stress markers to extend lifespan while improving metabolic health
ADF	16-week		KK and KKAy mouse model	Reduced blood glucose levels and preserved pancreatic β-cell function by modulating the hepatic AMPK/mTOR axis, effectively countering insulin resistance
	3-month		aged hypertensive rats model	Normalized blood pressure by restoring renin-angiotensin system balance and upregulating α-klotho expression
	22-month		aged male mice	Enhanced sexual behavior through coordinated peripheral and central regulation, increased muscle tryptophan uptake coupled with reduced brain serotonin signaling
	4-week		male and female mice	Promoted active coping behaviors by enhancing norepinephrine release within the locus coeruleus–medial prefrontal cortex pathway
	5-week		diabetes-prone mice model	Substantial alternative splicing events in skeletal muscle, differentially regulating metabolic pathways through altered enzyme activities and intracellular signaling, collectively optimizing energy utilization
	8-week		hypertensive stroke-prone rats (SHRSP)	Lowered blood pressure by reconstituting gut microbial communities, specifically increasing Lactobacillus abundance, and modulating bile acid metabolism
	16-week		Alzheimer's disease (AD) mouse model	Induced gut microbiota remodeling, and increased beneficial metabolites like 3-indolepropionic acid, while modulated ketone body and bile acid metabolism.
	10-day		mouse	Sensitized mice' leptin signaling in dorsal root ganglion neurons (evidenced by increased pSTAT3), promoted axonal regeneration after sciatic nerve and spinal cord injury via the cAMP–gene transcription pathway
	3-month		ischemic stroke rats model	Enhanced DNA damage repair through the USP18/SKP2 axis
	6-month		mice	Enhanced cardiac energy metabolism efficiency via AMPK activation and remodeled intracellular mitochondrial networks to support heart function
	12-week	12-week high fat diet feeding then change to 12-week high fat diet with ADF	rats	Alleviated high-fat diet-induced intestinal inflammation and metabolic dysregulation via the FXR/GLP-1/MC4R/PPAR-γ signaling axis
	4-week		mice	Enhanced synaptic plasticity through brain-derived neurotrophic factor signaling
5:2 Diet	5 weeks or 4 months or 7 months	5:2 diet or 6:1 diet	mice with NASH inducing diets (WD or CD-HFD)	Prevented NASH development as well as ameliorated established NASH and fibrosis without affecting total calorie intake and metabolome analyzes identified that peroxisome-proliferator-activated receptor alpha (PPARα) and glucocorticoid-signaling-induced PCK1 act co-operatively as hepatic executors of the fasting response
	4-week	4:3 diet	Parkinson's disease mice model	Enhanced autophagic clearance of toxic α-synuclein aggregates, reduced dopaminergic neurodegeneration, and improves motor function, while IF-associated molecules alleviate neuroinflammation in primary neurons

### 2.2. Metabolic regulation of intermittent fasting

Through its metabolic benefits, IF exerts systemic effects through sophisticated cross-organ coordination, thereby demonstrating protective or ameliorative potential across various NCDs.

In human trials, a 6-week randomized controlled trial (RCT) with 33 healthy young men illustrated IF's healthy effect. The participants assigned to the TRE group (16 h fasting per day) showed significant reductions in body weight and fat mass, along with increased basal fat oxidation, though no marked differences emerged in exercise-induced fat oxidation or other metabolic markers. These findings primarily position TRE as an effective approach to improve body composition [35]. Moreover, another RCT demonstrated that 6-month TRE (20 h fasting on three nonconsecutive days per week) yielded greater improvements in glycated hemoglobin and insulin sensitivity in type 2 diabetes patients compared to traditional CR [36]. Regarding ADF, 4-week ADF alone effectively counteracted obesity and enhanced metabolic function. When combined with dietary conversion (from high-calorie diet to normal chow diet), ADF produced optimal improvements in glucose tolerance, alongside effects linked to gut microbiota changes (including *Alistipes finegoldii*) and short-chain fatty acid (SCFAs) production, thus establishing a mechanistic basis for metabolic health interventions [37]. The practical applicability of IF extends to challenging populations such as shift workers. In a 24-week, three-arm trial that involved 250 overweight or obese shift workers, both the 5:2 diet (with two fasting days per week across shifts) and continuous CR led to significant weight loss and improved insulin resistance (measured by Homeostatic Model Assessment of Insulin Resistance (HOMA-IR)). Importantly, the 5:2 diet group achieved greater reductions in total cholesterol and LDL cholesterol, thus supporting the 5:2 diet as both a flexible and effective dietary strategy for this population [38]. Moreover, for a study among insulin-treated type 2 diabetes patients, the 12-week and 3-day per week 4:3 diet achieved sustained weight reduction over a two-year follow-up and demonstrated excellent safety [39]. The FMD, which was conducted for 5 consecutive days per month for 3 months, reduced body weight, trunk, and total body fat, lowered blood pressure, and decreased levels of insulin-like growth factor 1 (IGF-1). After 3 months, control diet subjects were crossed over to the FMD program, which resulted in a total of 71 subjects completing three FMD cycles. A post hoc analysis of subjects from both FMD arms showed that the body mass index, blood pressure, fasting glucose, IGF-1, triglycerides, total and low-density lipoprotein cholesterol, and C-reactive protein (CRP) were more beneficially affected in participants at risk for disease than in subjects who were not at risk [29].

Moreover, in numerous animal studies, the multidimensional regulatory effects of IF have been validated. In mice models of primary obesity and type 2 diabetes, a 16-week ADF intervention significantly reduced blood glucose levels and preserved pancreatic β-cell function by modulating the hepatic AMPK/mTOR axis, thus effectively countering insulin resistance [40]. The cardiovascular benefits are equally noteworthy. In aged hypertensive rats model, 3-month ADF normalized blood pressure by restoring the renin-angiotensin system balance and upregulating α-klotho expression [41]. Reproductive aging shows similar responsiveness. Male mice who commenced ADF at 2 months age achieved significantly higher reproductive success at 24 months (83% *vs*. 38%, *p* < 0.001). Mechanistic studies revealed that ADF enhanced sexual behavior through coordinated peripheral and central regulation: increased muscle tryptophan uptake coupled with reduced brain serotonin signaling [42]. Neurological benefits also manifest in stress resilience by enhancing norepinephrine release within the locus coeruleus–medial prefrontal cortex pathway; compared with ad libitum and acute fasting (fasting 24 h before test), 4-week ADF promoted active coping behaviors [43]. The synergy between IF and other interventions is particularly promising. When combined with physical exercise in rats, TRE (16 h fasting per day for 8 weeks) produced optimal outcomes in androgen regulation and muscle development, thus underscoring the value of integrated approaches [44]. IF's therapeutic reach significantly extends to degenerative conditions. In aged mice, a 10-week TRE (18 h fasting per day) enhanced motor coordination and muscle strength and increased myelin-associated glycoprotein expression, thus effectively decelerating neurodegenerative aging through myelin preservation [45]. In a high-risk Alzheimer's disease (AD) mice model, a 3-month TRE (18 h fasting per day) enhanced brain-derived neurotrophic factor signaling, thus constituting one mechanism through which fasting interventions ameliorate cognitive decline [46].

Interestingly, tissue-specific responses further illuminate IF's precision. In the diabetes-prone mice model, the TRE-exercise and ADF-exercise combination induced substantial alternative splicing events in skeletal muscle, thereby differentially regulating metabolic pathways through altered enzyme activities and intracellular signaling, thus collectively optimizing energy utilization. Conversely, adipose tissue showed more modest changes under the same conditions, thus suggesting that skeletal muscle possesses superior metabolic plasticity in responding to IF [47]. Systematic reviews corroborated this tissue selectivity, thus indicating that TRE preferentially targets adipose tissue to regulate energy balance, thereby promoting lipolysis and metabolic turnover and preserving lean mass and muscle integrity [48]. A narrative review concluded that IF supports neurogenesis and maintains brain plasticity by strengthening synaptic connections, improving signaling efficiency, and reducing neuroinflammation [49].

### 2.3. Modulation of gut microbiota and metabolic interactions by intermittent fasting

Urbanization has fostered dietary patterns characterized by processed foods and excessive antibiotic use, thereby significantly depleting gut microbial diversity and compromising its function. This dysbiosis substantially contributes to metabolic disorders and immune dysfunction, thus representing a key pathway in the development of NCDs [50]. As an established dietary regimen, IF demonstrates considerable potential for disease prevention and management through gut microbiota-dependent mechanisms.

The metabolic benefits of IF are intimately connected to its ability to remodel the gut ecosystem. A systematic review of 12 human studies on rheumatoid arthritis revealed that medical short-term fasting (7–10 days) significantly reduced the disease activity (measured by DAS28 scores) and inflammatory markers including CRP, while alleviating joint symptoms. These clinical improvements coincided with gut microbiota restructuring and enhanced intestinal barrier integrity [51]. An additional study that combined humans and animals on resistant hypertension further substantiated this connection, where a 2-week TRE (16 h fasting per day) in Resistant Hypertension (RH) patients and drug-resistant spontaneously hypertensive rats (SHRs) produced marked blood pressure reduction, accompanied by enriched *Akkermansia muciniphila* and *Adlercreutzia equolifaciens*. Crucially, fecal microbiota transplantation confirmed that IF-mediated microbial changes directly contribute to blood pressure regulation, alongside beneficial metabolite shifts including reduced Lipopolysaccharide (LPS) and Trimethylamine N-oxide (TMAO), and elevated SCFAs [52]. In spontaneously hypertensive stroke-prone rats (SHRSP), 8-week ADF substantially lowered blood pressure by reconstituting gut microbial communities, specifically increasing Lactobacillus abundance, and modulating bile acid metabolism [53]. A fecal metagenomic analysis revealed that mice with colorectal cancer (CRC) treated with 42-day FMD were enriched with *Bifidobacterium pseudolongum*, which promoted the differentiation of CD8+ T cells into memory cells by generating L-arginine, thereby synergistically demonstrating the anti-tumor effect of oral decisive therapies on colorectal cancer [54].

The gut-brain axis emerges as a pivotal pathway for IF-mediated neuroprotection. In the AD mice model, a 16-week ADF intervention induced comprehensive gut microbiota remodeling, thereby increasing beneficial metabolites such 3-indolepropionic acid while modulating ketone body and bile acid metabolism. These changes, coupled with an enhanced intestinal barrier function, collectively suppressed cerebral beta-amyloid deposition and significantly improved cognitive performance through gut-brain axis signaling [55].

### 2.4. Molecular mechanisms of if in regulating health effects

IF exerts its health effects through sophisticated molecular networks and metabolic pathways: through the moderate stress of energy metabolism during fasting, the body can produce moderate stress to regulate the its metabolic ability. The main mechanism involves the regulation of hormone and enzymatic secretion, autophagy regulation, and anti-inflammatory effects.

IF regulates health through hormonal and enzymatic pathways. In peripheral nerve repair, ADF for 10 days sensitizes mouse leptin signaling in dorsal root ganglion neurons (evidenced by increased pSTAT3), thereby promoting axonal regeneration after sciatic nerve and spinal cord injury via the cAMP–gene transcription pathway, thus revealing leptin's non-canonical role in neural repair [56]. In ischemic stroke rats model, ADF for 3 months enhanced DNA damage repair through the USP18/SKP2 axis, which inhibited β-catenin ubiquitination and thereby delayed neurodegenerative progression [57]. In a dextran sodium sulfate (DSS) induced colitis mice model, a 36-day TRE (16 h fasting per day) could reduce intestinal oxidative stress by decreasing intracellular reactive oxygen species while enhancing antioxidant enzymes including superoxide dismutase (SOD) and glutathione peroxidase (GSH-Px) [30]. A 4-week TRE (18 h fasting per day) additionally reversed high-fat diet-induced males and females mice memory impairment by restoring normal cortex-hippocampus neural coupling, which is linked to rebalanced glucocorticoid receptor Ser134/Ser226 phosphorylation [58]. The 5:2 diet prevents non-alcoholic steatohepatitis (NASH) development and ameliorates established NASH and fibrosis without affecting the total calorie intake. Combined proteome, transcriptome, and metabolome analyses identified that peroxisome-proliferator-activated receptor alpha (PPARα) and glucocorticoid-signaling-induced PCK1 act co-operatively as hepatic executors of the fasting response [59]. A 42-day FMD in combination with chemotherapy in a mice model showed that FMD delayed the progression of breast cancer and melanoma by increasing the number of common lymphoid progenitor cells and cytotoxic CD8+ tumor-infiltrating lymphocytes in the bone marrow, alongside down-regulating the expression level of the stress response enzyme heme oxygenase-1 [60]. In a mice model of hormone receptor-positive breast cancer, FMD between 35 and 60 days enhanced the efficacy of tamoxifen and fulvestrant by reducing circulating insulin-like growth factor-1, insulin, and leptin levels and inhibiting AKT-mTOR signaling via upregulation of EGR1 and PTEN. Meanwhile, in estrogen-treated patients with hormone-receptor-positive breast cancer, an average 6-month FMD elicited metabolic changes that were similar to those in mice, including reduced levels of insulin, leptin, and IGF1 [61].

In autophagy regulation effects, a 4-week dawn-to-dusk (15 h fasting per day) TRE upregulated key autophagy genes (LAMP2, LC3B, and ATG5) while improving metabolic and inflammatory markers in overweight individuals [62]. Additionally, AI-driven analyses identified spermine as a core mediator of fasting-induced autophagy and longevity. By promoting the hydroxybutyl lysine modification of eIF5A, spermine facilitates metabolic reprogramming, thus revealing novel connections between interferon signaling, autophagy, and the organismal lifespan [63]. Moreover, animal studies demonstrated that cardiovascular benefits similarly traced to mitochondrial adaptations, where 6-month ADF and TRE (12–16 h fasting per day) in mice models both enhanced the cardiac energy metabolism efficiency via AMPK activation and remodeled intracellular mitochondrial networks to support heart function [64]. Research indicates that TRE reverses brain pathology in AD mice model by restoring circadian autophagy, a mechanism linked to Bmi1 upregulation, which suppresses downstream targets including Nfatc1, Stra6, and Tlr2 while activating Npy. The PPAR signaling axis connects TRE-mediated benefits to circadian regulation, thus revealing novel therapeutic targets for AD [46].

The anti-inflammatory properties of IF constitute another major therapeutic dimension. For humans, in obese type 2 diabetes patients, a 3-week TRE (16 h fasting per day) reduced the LDL cholesterol, total cholesterol, and leptin levels while elevating β-hydroxybutyrate levels. This ketone body subsequently modulates inflammatory responses in macrophages and T cells, thus attenuating systemic inflammation [65]. A 72-hour fasting triggers extensive transcriptomic and proteomic changes in white blood cells, thereby upregulating autophagy pathways while downregulating apoptosis. This enhances leukocyte viability and stimulates neutrophil degranulation and cytokine secretion, collectively strengthening the innate immunity [66]. In rats model, a 12-week high fat diet feeding followed by a change to a 12-week high fat diet with ADF could alleviate high-fat diet-induced intestinal inflammation and metabolic dysregulation via the FXR/GLP-1/MC4R/PPAR-γ signaling axis [67]. Anti-neuroinflammation appears central to IF's benefits, thus warranting expanded gut-brain axis investigations. Two reviews demonstrated that in a neurodegenerative model, IF reduced neuroinflammation by lowering pro-inflammatory cytokines (TNF-α, IL-6) and promoting ketogenesis, thus ameliorating anxiety and depressive behaviors [68],[69]. A Parkinson's disease (PD) mice model demonstrated that a 4-week fasting for 3 days per week intervention enhanced autophagic clearance of toxic α-synuclein aggregates, reduced dopaminergic neurodegeneration, and improved motor function, while IF-associated molecules alleviated neuroinflammation in primary neurons, thus supporting non-pharmacological interventions in PD [70].

### 2.5. Multi-omics insights into intermittent fasting

The metabolic mechanisms underlying IF operate through complex, multi-level pathways, thus making comprehensive multi-omics analyses essential to fully elucidate its health impacts. Circadian dimensions emerge as particularly crucial, where a 3-week TRE (12 h fasting per day) in mice model synchronized mitochondrial metabolic rhythms through clock genes such as CLOCK/BMAL1, thus resulting in 100% improvements in exercise endurance [71]. Integrated metabolomic and metagenomic analyses demonstrated that a TRE that lasted more than 22 months (16 h fasting per day) improved metabolic homeostasis by enriching SCFA-producing bacteria (including *Akkermansia muciniphila*) and regulating key poly-unsaturated fatty acid (PUFA) metabolic pathways [72]. For a 7 week TRE (15 h fasting per day) in a mice model, RNA sequencing and a metabolomics analysis of samples collected every 2 hours over a 24-hour period revealed that 80% of genes exhibited differential expression or rhythmic alterations induced by TRE in at least one tissue. TRE enhanced gene expression rhythmicity in most tissues, phase-coordinated the expression of anabolic and catabolic genes, downregulated genes related to inflammatory signaling and triglyceride metabolism, and upregulated genes associated with RNA processing, protein folding, and autophagy. These effects collectively mediate multi-tissue nutrient metabolism reprogramming, enhance metabolic flexibility, and improve hepatic metabolism [73]. Metabolomic evidence indicates that CR combined with TRE (20 or 16 hours fasting per day) for 45 weeks significantly alters serum metabolic profiles in aged female mice, thereby reducing oxidative stress markers to extend the lifespan while improving the metabolic health [74]. Moreover, the neurological benefits of IF find mechanistic support in metabolomic data, where a 4-week ADF in mice model revealed enhanced synaptic plasticity through brain-derived neurotrophic factor signaling [43].

The impact of IF on health is related to a variety of molecular mechanisms and metabolic pathways, and its impact range is very wide, which needs continuous research and exploration and long-term intervention analyses. While multi-omics data have powerfully illuminated IF's complex regulatory networks, the sheer volume of information presents significant analytical challenges. This deluge of multidimensional data urgently requires advanced computational frameworks capable of integrating and interpreting these complex datasets, thus enabling researchers to more clearly understand IF's multifaceted biological functions.

## 3. Potential applications of artificial intelligence in intermittent fasting research

**Figure 3.**
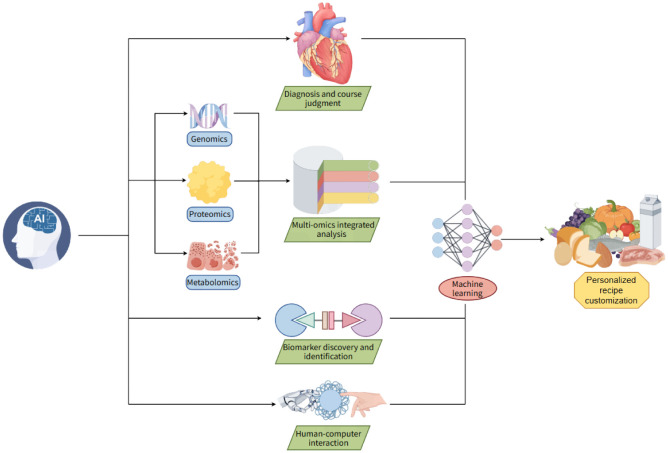
Introduction of artificial intelligence promoting personalized precision medicine of Intermittent Fasting. AI assists scientific research and clinical data analyses, which can promote the progress of disease and course diagnosis, the integrated analysis of multi-omics data, mining, and matching of molecular targets and markers, and the interactive research between multidisciplinary personnel and medical AI. The data obtained can feed back to the machine, and then intelligently order accurate personalized recipes through deep learning analyses.

The rapid advancement of AI is fundamentally transforming medical practices across numerous specialties, thereby significantly enhancing the diagnostic accuracy, therapeutic precision, and operational efficiency. This review examines the current applications and progress of AI technologies in various medical domains, with particular emphasis on how AI can facilitate the development of personalized IF interventions within precision medicine frameworks. A preliminary conceptual overview of this integration is presented in Figure 3.

### 3.1. Assisted diagnosis and precise disease staging

Healthy dietary patterns are universally recognized as cornerstone strategies for chronic disease prevention and management [7],[75], with IF increasingly being validated for mitigating various chronic conditions [76]; however, effective implementation requires precise metabolic disease staging. Conventional diagnostic paradigms primarily rely on the clinician's interpretation of multimodal data (medical history, physical examinations, imaging, and laboratory parameters). In contrast, AI could support quantitative diagnostics by detecting subtle phenotypic variations and risk factors embedded within complex multidimensional signals. Through advances in machine learning (ML) and DL, AI algorithms can process raw, unstructured biometric signals and images, thus uncovering novel biomarkers inaccessible to conventional diagnostics, thereby continuously refining chronic disease assessment precision. The Human Phenotype Project (HPP) exemplifies this potential by employing self-supervised learning on dietary records and continuous glucose monitoring data. The HPP developed AI models that outperformed existing methods in predicting disease onset [77]. Additionally, AI augments diagnostic capabilities through the integration of multi-source cognitive data. Through an analysis of non-traditional biomarkers including speech patterns, eye movements, and digital behavioral metrics, AI provides supplementary cognitive function insights, constructs comprehensive disease profiles, enhances diagnostic accuracy, and deepens the understanding of pathological mechanisms [78]–[80].

AI's diagnostic potential has been explored across multiple clinical domains. Algorithms that leverage electronic health record data can non-invasively quantify atherosclerosis, map continuous mortality risk, and identify underdiagnosed individuals with coronary artery disease [81]. Cardiovascular innovations include AI-assisted sonographer systems for cardiac function evaluation [82], and intelligent Electrocardiogram-based (ECG-based) assessments of left ventricular ejection fraction [77]. In hepatology, AI-enhanced digital pathology detected early degenerative septal changes in liver disease [83], and ML-based histological scoring predicted hepatic hemodynamic changes in cirrhotic metabolic dysfunction-associated steatohepatitis (MASH) patients [84],[85]. In diabetes research, AI-driven models (integrating tongue features or fundus images) achieved robust diagnostic performance (achieving area under the receiver operating characteristic (AUROC) 0.924) [86],[87]. In transplantation medicine, AI integration of clinical, demographic, and immunological data showed promises to improve rejection monitoring, personalize immunosuppression, and optimize patient outcomes [88]. Overall, AI is gradually refining disease diagnosis paradigms and dynamic analysis capabilities. Through continuous data integration and model training, AI systems may enhance the simultaneous analysis of massive multivariate indicators, thereby translating previously unquantifiable data patterns into detection of subtle pathological changes, which supports the advancement of precision medicine across healthcare domains.

Accurate disease diagnoses coupled with early lifestyle interventions have emerged as a crucial prevention and treatment strategy for numerous NCDs. Importantly, IF's metabolic regulatory mechanisms stand to be significantly enhanced through AI assistance. A review revealed that ML has already been employed to analyze the effects of TRE on circadian rhythmicity [88]. Another study harnessed the power of AI along with big data collected from a large cohort of healthy individuals. This personalized nutrition project showed that glycemic responses to identical foods varied among individuals and suggested that dietary approaches that aim to control and lower blood glucose levels may have to be individually adjusted [89]. An AI-driven personalized meal planning system was developed to meet the healthy dietary needs of people with chronic diseases such as cardiovascular disease and diabetes. The meal plans generated by the system performed well in nutritional optimization (Prerow Value above 0.8) and user preference fit [Technique for Order Preference by Similarity to Ideal Solutionb (TOPSIS) score 0.5227]. Moreover, it solved the problems of the lack of personalization and full consideration of health constraints and preferences in existing dietary planning tools; however, there are still limitations such as the lack of dynamic updates [90]. The above studies show that accurate disease diagnoses are the key factors of diet planning to prevent and treat diseases, and the main area of AI assistance.

AI has shown significant technical advantages and development potential in the field of medical diagnoses, especially in pattern recognition and real-time decision support of multimodal data, such as imaging, genomics and clinical text. However, the existing systems still have limitations in cross-population generalization ability, rare disease subtype recognition, and complex comorbidity scenarios. Prospective clinical trials and multicenter studies are needed to verify their real-world applicability, which also determines that AI should be located as an auxiliary tool rather than an independent diagnostic agent. In this context, medical experts still need to take the lead in clinical process design, data quality control, and the evidence-based integration of AI outputs to build a precision diagnosis and treatment system with human-computer collaboration.

### 3.2. AI empowering in mechanistic studies of intermittent fasting

AI plays an important role in exploring multi-dimensional biomarkers and is one of the key technical means to study the IF of complex metabolic networks. For instance, in anti-infective drug discovery, AI systems can efficiently navigate complex molecular databases, thereby leveraging ML methods to primarily identify novel compounds or repurpose existing drugs given the immense chemical space of drug-like small molecules (approximately 10⁶⁰ [91]). In neurodegenerative disease research, DL architectures such as CNNs can effectively identify characteristic neuroimaging abnormalities in AD. These models enable the automated detection of amyloid plaques and neurofibrillary tangles while improving diagnostic reproducibility [92]–[94].

The integration of AI in multi-omics analyses provides a new analytical perspective for the discovery of metabolism-related biomarkers. By leveraging multimodal biomedical data, it deciphers complex associations between cross-omics datasets and uncovers potential patterns. DL architectures that integrated radiomic and pathomic data demonstrated potential in predicting biomarkers associated with a patient's responses to immunotherapy [95]–[97]. Extending this paradigm, natural language processing models trained on radiology text reports have shown improved an accuracy in predicting the best overall response and progression-free survival in non-small cell lung cancer patients following PD-1 blockade therapy [98]. ML approaches now routinely apply to novel biomarkers and construct dynamic association models between these biomarkers and disease progression, thereby providing support for earlier interventions and the development of more personalized therapeutic strategies [99]–[101].

AI exhibits notable potential in integrating multi-omics data to assist in elucidating the molecular mechanisms of IF. Additionally, its comprehensive mining of existing large-scale datasets enables the identification of both previously overlooked and novel biomarkers, thereby offering critical insights to develop more precisely individualized dietary or therapeutic interventions. One research team proposed to use ML to analyze the unique chemical fingerprints derived from the metabolomic study of cellular metabolic processes and to explore the potential of neural networks in assessing nutritional biomarkers, predicting BMI, and discovering dietary patterns. They developed a deep neural network model for BMI classification based on biochemical characteristics and a classification model for nutritional characteristics [102]. Supervised ML models have been successfully applied to analyze diverse structured and unstructured datasets that encompass nucleic acids, proteins, carbohydrates and cellular phenotyping data and to effectively identify key molecules and regulatory networks that underlie host-pathogen interactions and immune responses. [103]–[105]. By integrating FooDB and DrugBank, the FDMine analysis framework was developed, which constructs similarity feature and association prediction algorithm models to analyze food-drug interactions (FDIs) based on known biological pathways or pharmacological effects. This framework identifies novel FDIs with anti-inflammatory potential, such as β-adrenergic antagonists and dietary components such as eugenol and methyleugenol, which synergistically enhance the antihypertensive efficacy. These findings provide a new theoretical basis for the study of the synergistic mechanism of dietary intervention and drug treatments [106].

Through ML and DL algorithms, AI can efficiently analyze complex associations among multi-dimensional data. When combined with methods such as feature screening and pathway enrichment analyses, it significantly improves the efficiency of the potential biomarker identification. However, the biological significance and clinical value of AI-predicted results still require systematic evaluations through actual experiments and prospective clinical trials. Currently, AI primarily serves as an auxiliary tool: the credibility of its outputs relies on supplementary experimental verification, and domain experts play a central role in feature engineering, model optimization, and result interpretation.

### 3.3. AI promotes precision medicine and personalized application of Intermittent Fasting

Unlike targeted pharmacological therapies, dietary interventions operate through systemic metabolic modulation rather than addressing specific disease etiologies. By influencing the body's overall metabolic landscape, these interventions enhance innate regulatory mechanisms to prevent and manage disease. This multifaceted nature introduces considerably more variables than conventional diagnostics, thus necessitating sophisticated analytical approaches. Consequently, diverse AI algorithm models developed across research teams and databases collectively provide invaluable reference data to formulate evidence-based dietary patterns.

In clinical nutrition, AI is revolutionizing precision through large-cohort analyses. These technologies introduce novel paradigms of precision and personalized nutrition, thus enabling individuals to receive dietary recommendations optimally tailored to their unique physiological characteristics and real-life contexts [107]. Several pioneering systems demonstrate this potential:

1. ML-assisted calories to satiation genetic risk scoring (CTSGRS) effectively predicts individual responses to calorie intake. In randomized trials, the participants with elevated CTS or CTSGRS exhibited significantly greater weight loss with pharmacological intervention, thus highlighting its utility for personalized obesity management strategies [108].

2. The Microbiome-based Nutrient Profile Corrector (METRIC) employs DL to rectify random errors in self-reported dietary assessments (from 24-hour recalls or food records) using gut microbiome composition data. This innovation enables a more accurate evaluation of personalized dietary impacts [109].

3. McMLP DL models predict metabolite responses to dietary interventions based on gut microbiome profiles. Across both synthetic data from microbial consumer-resource models and six human dietary intervention studies, McMLP outperformed random forest and gradient boosting regression, while a sensitivity analysis revealed food-microbe-metabolite interactions, thus informing microbiota-based personalized nutrition strategies [110].

4. eXtreme Gradient Boosting (XGBoost) models predict microbial burden from fecal species and gene abundance data, thereby identifying target species and their associations without laboratory testing, thus providing valuable tools for precision medicine applications [111].

5. Ontology-based Type 2 Diabetes Diet Decision Support System (OnT2D-DSS) exploits clinical expert knowledge formalized as a domain ontology to identify a patient's phenotype and potential comorbidities, thus providing personalized MNT recommendations for macro- and micro-nutrient intake [112].

In designing personalized fasting regimens, AI-powered bibliometric tools such as CiteSpace identified crucial IF research domains including circadian biology, metabolic diseases, and gene regulation [113]. By integrating multidimensional individual data, genetic predispositions, lifestyle patterns, and health status, AI enables the formulation of precise fasting protocols that enhance both safety and efficacy. For instance, research confirms that a 30-day TRE (16 h fasting per day) ameliorates immunosenescence and promotes gut microbiota health by modulating CD4⁺ Treg cells and increasing *Akkermansia muciniphila* abundance [114]. Current AI models already demonstrate high-quality recommendations for diabetic IF protocols, though continued professional refinement remains essential. Future directions include mobile health platforms and dynamically adaptive fasting plans [115]. Further investigation into how fasting duration and individual health status modulate outcomes will be crucial to advance the clinical translation of personalized fasting protocols.

### 3.4. Interdisciplinary collaboration and human-computer interaction development

The integration of AI is bringing unprecedented opportunities to IF research, with several advanced technologies demonstrating considerable promise. In medical imaging, CNNs, a type of DL algorithm with exceptional pattern recognition capabilities, facilitate advances in neuroimaging analyses. When combined with magnetic resonance imaging (MRI) and trained on extensive datasets of brain scans, CNNs can accurately identify subtle structural changes characteristic of early neurodegeneration, including localized atrophy and white matter abnormalities. This capability enables an earlier diagnosis and a more precise prognosis of AD [116]. Simultaneously, generative AI (genAI) represents a notable shift in analytical flexibility. Unlike traditional ML methods, genAI seamlessly adapts to multiple data patterns, thus capturing and centralizing relevant information flows. Its user-friendly interface enhances usability while allowing clinicians a degree of customization, thus showing significant potential in dynamic human-computer interaction scenarios [117]. Practical applications of AI in this field continue to expand: although cardiac ultrasound devices have become increasingly portable, their operation and interpretation traditionally required specialized expertise. Now, DL-based guidance systems enable less experienced operators to acquire diagnostic-quality images, thus broadening the access to advanced cardiac assessments [118]. Furthermore, multimodal AI systems can integrate diverse data sources, including genomic data, imaging results, and electronic health records, to establish precision medical models for complex conditions such as bipolar disorder, thus enabling sophisticated patient stratification and intervention guidance [119]. A clinical validation study conducted in Ghana evaluated the accuracy of a Food Recognition Assistance and Nudging Insights (FRANI) mobile terminal AI dietary assessment tool. The study included 36 female adolescents aged 12–18 years, with a parallel control trial comparing it to the weighing method and 24-hour dietary recall method. The results showed that the nutrient intake measurements from the FRANI tool were comparable to those of traditional dietary assessment methods, thus reflecting the feasibility of AI in dietary prediction [120].

Despite these advances, AI-driven personalized nutrition research faces significant challenges, with ethical considerations being particularly salient. The highly sensitive nature of the involved data necessitates stringent protocols for collection, storage, and usage that prioritize privacy protection and cybersecurity. To address current research limitations, future efforts should prioritize interdisciplinary collaborations. Integrating dynamic multi-omics data with AI predictive models will support the development of more comprehensive evaluation systems and real-time feedback-regulated fasting modulation platforms.

## 4. Challenges and future perspectives

Current research on the mechanisms through which IF regulates human health and homeostasis continues to advance rapidly. Thereby leveraging its robust capabilities in data integration and in-depth analysis, AI is increasingly emerging as a pivotal driver of progress in precision nutrition and health. Through deep integration of multidimensional health data, including key variables such as sex differences and fasting durations, AI holds promise to construct more precise and efficient individualized dietary regimens, thus offering novel strategies for chronic disease prevention and public health promotion. By summarizing the existing studies on the health effects of IF, this review analyzed the potential of clinical reference of IF, and found that there were some problems such as the lack of long-term experiments and the poor comparability of existing studies. At the same time, it found the potential ability of AI to solve the problems of IF, such as the early diagnosis and timely inclusion of dietary intervention of IF. It aids in multi-omics and biomarker analyses for the mechanistic study of IF. Most importantly, AI makes it possible to formulate portable, personalized and precise dietary strategies.

IF research faces three major challenges: data heterogeneity, where variations in CR intensity and complex interactions between epigenetic factors and environmental exposures limit the reproducibility of findings; technical constraints, as existing AI models demonstrated a limited efficiency in extracting hierarchical features from multi-omics data and their interpretability scores remain below the threshold required for clinical applications, thus hindering their decision-making utility; and clinical translation barriers, characterized by a severe scarcity of long-term tracking data with prospective cohorts that exceed five years, thus representing only 12% of available studies, which impedes the reliable assessment of IF's long-term effects. These obstacles intersect with broader challenges in AI clinical integration, where gaps in infrastructure, reimbursement pathways, and regulatory frameworks collectively constrain the adoption of AI-based applications in routine pathology. The future advancement of AI in digital pathology ultimately depends on establishing sustainable implementation models through concerted efforts by all stakeholders to integrate these technologies into standard clinical practice [121].

To address these challenges, we propose a strategic three-pillar framework that begins with the development of FAIR-compliant omics databases to resolve data heterogeneity through enhanced findability, accessibility, interoperability, and reusability, thus enabling high-quality data sharing and integration. The second pillar involves building a sophisticated computational architecture that integrates transfer learning and knowledge graphs to advance the multi-omics feature extraction and model interpretability, thus ultimately enhancing AI's usability and transparency in clinical settings. Finally, we recommend implementing precision intervention trials that bridge nutritional science with clinical medicine to systematically accumulate long-term efficacy data, thereby establishing a closed-loop translational pipeline from mechanistic research to personalized applications.

Future IF research should prioritize the development of efficient cross-omics integrated analysis platforms aimed at enhancing the model's generalizability, breaking down data silos, and deepening our understanding of mechanisms and intervention optimization. Concurrently, strengthening interdisciplinary collaboration by integrating expertise from nutritional science, bioinformatics, and clinical medicine will be essential to advance the precise design and clinical translation of IF interventions. This comprehensive approach will ultimately transform IF from a generic dietary strategy to a precisely calibrated therapeutic intervention, thus positioning AI as the cornerstone of next-generation precision nutrition and paving the way for truly personalized preventive health strategies that can adapt to individual physiological responses and long-term health trajectories.

## Use of AI tools declaration

The authors declare they have used Artificial Intelligence (AI) tools in the creation of this article.
